# Antihypertensive activity, toxicity and molecular docking study of newly synthesized xanthon derivatives (xanthonoxypropanolamine)

**DOI:** 10.1371/journal.pone.0220920

**Published:** 2019-08-15

**Authors:** Omprakash Goshain, Bahar Ahmed

**Affiliations:** Department of Pharmaceutical Chemistry, Faculty of Pharmacy, Jamia Hamdard University, New Delhi,India; Aligarh Muslim University, INDIA

## Abstract

**Context:**

Xanthone derivatives have been reported to possess a wide range of biological properties. In effort to search new effective antihypertensive compounds, we have synthesizednovel xanthone derivatives (xanthonoxypropanolamines) and got patent for these compounds (The Patent Office, Government of India, S. No.: 011–016308, Patent No.: 250538).

**Objective:**

In the present work, we attempted to establish the antihypertensive activity, toxicity and molecular docking study forthese newly synthesized compounds (1a, 1b and 2).

**Materials and method:**

The preliminary antihypertensive screening was performed by administering synthesized compounds and standard drugs intraperitonially and orally into wistar rats. The change in systolic, diastolic and the mean blood pressure before and after the treatment of the drugs was measured on a Digital LE-S100 Blood Pressure Meter by Tail-cuff method non-invasively. Toxicity studies were carried out after oral administration of synthesized compounds to rats at doses of 25, 50, and 100mg/kg. The serum samples were tested for different toxicity parameters such as liver function test, kidney function test etc. The docking simulations of all the compounds were performed using Maestro, version 9.4 implemented from Schrodinger software suite.

**Results and discussion:**

The result showed that the compound 1a, 1b and 2 have greater antihypertensive activity with almost equal or less toxicity profile in comparison to standard drug Propranolol and Atenolol. The docking score for the compound **1b** was found **-9.1** while for compound **1a** and **2** were found **-8.7** and **-8.6** respectively.

**Conclusion:**

These novel compounds i.e. 1a, 1b, and 2 have greater antihypertensive activity in comparison to standard drugs Propranolol and Atenolol. All these compounds do not have any toxicity.

## Introduction

Xanthones represent a large group of heterocyclic compounds including natural, semi synthetic and totally synthetic structures. Chemically, xanthonic nucleus corresponds to dibenzo-γ- pyrone. Xanthone molecules, having a variety of substituents on the different carbon of the nucleus, constitute a group of compounds with a broad spectrum of biological activities. Xanthone derivatives have been reported to possess a wide range of biological properties including antimalarial [1], anticonvulsant [2], anticancer [3], antidiabetic [4], antioxidant [5], anti-inflammatory [6], and antihypertensive [7, 8] activities. In effort to search new effective antihypertensive compounds, we have synthesized eleven xanthone derivatives (xanthonoxypropanolamines) and got patent for these compounds [9]. In the present work, we attempted to establish the antihypertensive activity and toxicity of these compounds (1a, 1b and 2)and performed exhaustive preliminary antihypertensive screening study and different toxicity study including lever function test, kidney function test, cardiac function test, lipid function test, Acetylcholine esterase, glutathione, catalase, superoxide dismutase (SOD) estimation and Thiobarbituric acid reactive substances (TBARS) Assay. The preliminary antihypertensive screening data of these compounds showed excellent antihypertensive profile. Different toxicity test data were compared with standard drug propranolol and atenolol. It was observed that these compounds are almost as safe as propranolol and atenolol.

Molecular docking, in the field of molecular modeling, is a method which gives best fit orientation of a ligand (drug) to their target molecules (receptors) by generating different score for different orientation. These score is known as docking score. More negative value of docking score indicates better fit orientation [10]. In order to strengthen the preliminary antihypertensive screening results, here we have also reported the docking score of all the three compounds and compared with standard drug propranolol.

**Fig 1:**
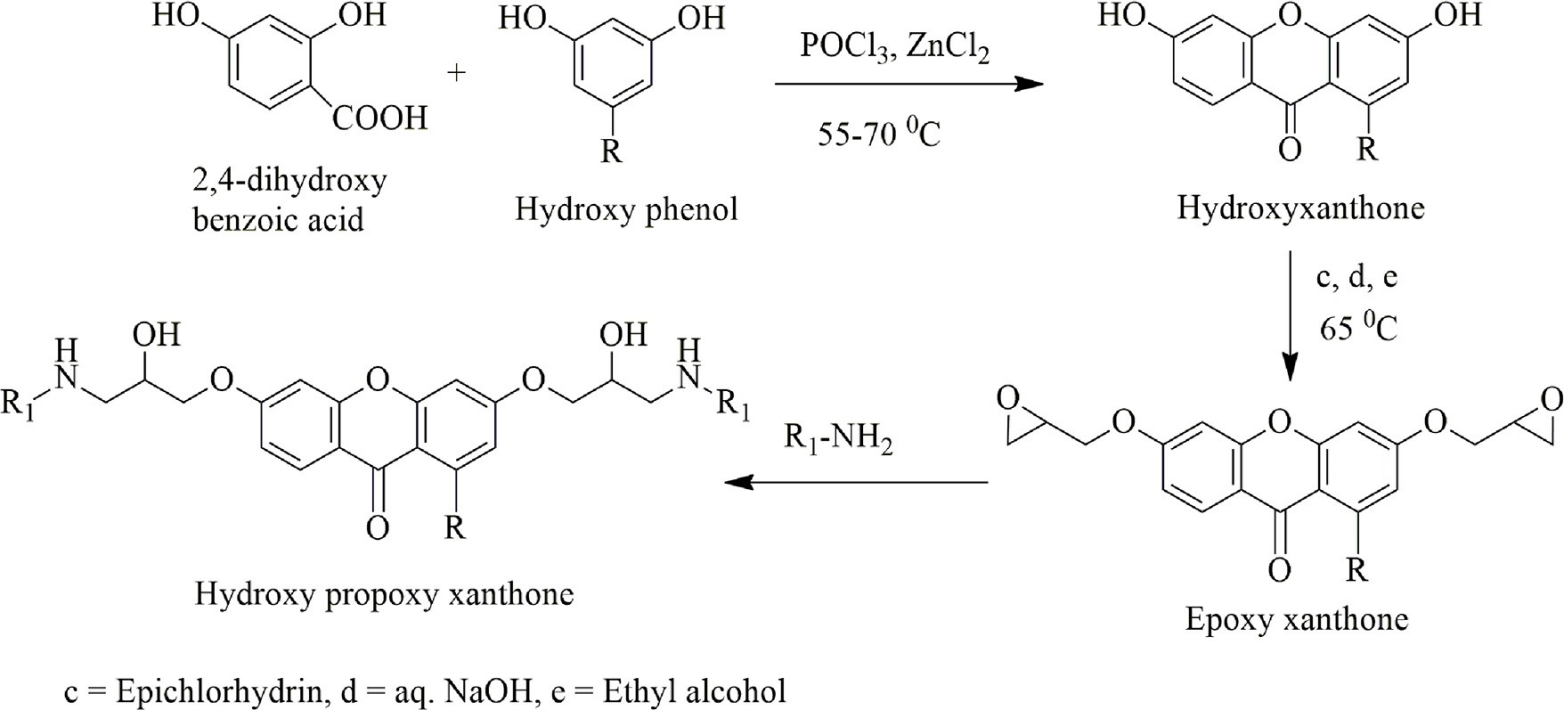
Synthesis of xanthonoxypropanolamines. **1a**; R = H, R_1_ = CH_2_CH_2_CH_3_ **1b**; R = H, R_1_ = CH (CH_3_)_2_ **2**; R = OH, R_1_ = CH (CH_3_)_2_.

The hydroxyl propoxy xanthones (xanthonoxypropanolamines) were synthesized by using general route for the synthesis of xanthonoxypropanolamines as shown in the Fig 1. The appropriate phenols were reacted with epichlorhydrin in the presence of alkali and ethanol to produce their epoxy derivatives. Ring opening of epoxides was occurred by refluxing in ethanol to get the aryloxypropanolamines derivatives.

## Materials and methods

The melting points were determined in one side sealed melting point capillary tubes using Thomas Hoover melting point apparatus and are uncorrected. The purity of the compounds was checked by thin layer chromatography (TLC) with solvent system benzene: methanol (8:2), using iodine vapours as visualizing agents and R_f_ values were calculated. Infra red spectra were determined on JASCO FT/IR-5300 Infrared Spectrophotometer by KBr disc method. ^1^H-NMR (300 MHz) and ^13^C-NMR (75 MHz) spectral studies were done on JEOL AL FTNMR Spectrophotometer using chloroform as solvent. The MS data were recorded on a Quattro micro API Waters’s mass spectrometer/ method—ESI-MS.

### Chemistry

#### Synthesis of novel xanthonoxypropanolamines [11]

**General Procedure**

The appropriate phenols (i.e. heterocyclic aromatic compounds containing hydroxyl groups) were condensed with epichlorhydrin in the presence of alkali and ethanol to obtain their epoxy derivatives. Ring opening of epoxides with various amines was achieved by refluxing in ethanol to afford the aryloxypropanolamines derivatives.

**Synthesis of di and tri-hydroxy xanthones**

A mixture of *o*-hydroxy benzoic acid or 2, 4 dihydroxy benzoic acid, a phenol (such as catechol, pyrogallol, resorcinol, etc.), freshly fused zinc chloride (ZnCl_2_) and phosphorous oxychloride (POCl_3_) was heated on a water bath or on heating mantle (maintaining the temperature 55-70°C) for 1.5–2 hr, cooled and poured into ice water carefully in order to avoid polymerization (Grover et al., 1955). The product was filtered off, washed with 2% sodium hydrogen carbonate solution (if needed) and water, dried and crystallized from suitable solvents. The recrystallization of compounds from appropriate solvents furnished analytically pure products.

**Preparation of epoxy compounds (3-aryloxy-2, 3-epoxy propane) from the respective hydroxyl-xanthones**

Epichlorhydrin was added to a solution of hydroxyl xanthones in an aqueous ethanol containing sodium hydroxide. The reaction mixtures were heated under reflux at 60-75°C for two hours with continuous stirring. The mixtures for each respective xanthones were stirred for further three hours at room temperature. The products were poured into ice water, the oily layer was separated out and concentrated under reduced pressure (or aqueous layer was decanted after keeping overnight in a refrigerator and was left in open air for evaporation up to dryness). The epoxy compounds, wherever required, were further purified by silica gel column chromatography using benzene: methanol (80:20) as eluent.

**Preparation of [(alkyl/aryl-amino)-2-hydroxy-propoxyl]–xanthones**

The prepared epoxy derivatives of xanthones, in absolute alcohol, were added slowly in large excess of different type of alkyl/aryl amines. The molar ratio of amines and epoxides were generally taken as (500–100:1). The reaction mixtures were refluxed with stirring at least 17 to 24 hrs (in some cases up to 2–3 days) at 50-65c. The mixtures were further stirred under reflux at room temperature for 1–2 days, if needed. The products were, then, subjected to evaporate in the vacuum to remove the remaining amines. The resulting amino-alcohols were purified and recrystallized from appropriate solvents.

#### Spectral data

**3, 6-Di–[3-(n-propyl-amino)-2-hydroxy-propoxy] xanthone (1a)**: Obtained as jelly solid (soluble in water and chloroform), reddish brown. m.p. 103–104°C, R_f_: 0.32 [Benzene: Methanol (8:2)], yield: 71.6%. UV (λ max CH_3_OH): 304, 280, 234, 224, 215 nm. IR: nmax (KBr): 3500–3450 (OH, NH), 2950 (CH_3_), 2807 (CH_2_), 1688 (C = O), 1550 (N-H and C = C merged), 1276 (N-H), 1221(C-O, phenolic), 1156, 1047 (C-O, alcoholic), 743 (C-H, aromatic), 626 cm-1. ^1^H NMR: (CDCl_3_, 300 MHz) δ 8.75 (2H, *d*, J = 8.0 Hz, H-1, H-8), 8.55 (2H, *d*, J = 2.0 Hz, H-4, H-5), 7.96 (2H, *dd*, J = 2.0, 8.0 Hz, H-2, H-7), 4.86 (4H, *m*, -OCH_2_-1′ and -OCH_2_-1″), 3.39 (2H, pentet, W1/2 = 17.0 Hz, -CH-2′ and CH-2″), 2.83 (4H, br*m*, -CH_2_-3′ and -CH2-3″), 2.46 (2H,*m* N-H- 4′, and N-H-4″), 1.58 (8H, *m*, -CH2-CH_2_-5′, 6′ and -CH_2_-CH_2_-5″, 6″), 0.87 (6H, *t*, J = 6.0Hz, -CH_3_-7′ and–CH_3_-7″). TOF MS ES+ (70 ev): *m/z* 458 (M+, C_25_H_34_ N_2_O_6_) (12), 403 (species -a) (7), 402 (species-b) (50), 358 (species-c) (18), 294 (species-d, base peak) (100), 273 (species-e) (35), 185 (species-f) (72).

**3, 6-Di–[3-(iso-propyl-amino)-2-hydroxy-propoxy]–xanthone (1b)**: Recrystallized from ethanol, soluble in water and ethanol. m. p. 101–102°C, Needle shaped long, white-reddish crystals. R_f_: 0.33 [Benzene: Methanol (8:2)] yield: 74.5%. UV (λ max CH_3_OH): 305, 296, 236, 222, 214, 211 nm. IR: nmax (KBr): 3298 (OH), 2977 (CH_3_), 2850 (CH_2_), 1651 (C = O and N-H), 1514 (C = C), 1450, 1367, 1225 (N-H), 1155 (C-O, phenolic), 760 (C = C), 566 cm-1. ^1^H NMR: (DMSO-d6, 300 MHz): δ 8.75 (2H, *d*, J = 8.0 Hz, H-1, H-8), 8.55 (2H, *d*, J = 2.0 Hz, H-4, H-5), 7.96 (2H, *dd*, J = 2.0, 8.0 Hz, H-2, H-7), 3.88 (4H, *brs*, -OCH_2_-1′ and -OCH_2_-1″), 3.24 (2H, pentets, W1/2 = 15.8 Hz, -CH-2′ and CH-2″), 2.80 (4H, *m*, -CH_2_-3′ and -CH_2_-3″), 2.0 (2H, *s* N-H- 4′, and N-H-4″), 1.84 (2H, septet, W1/2 = 17.5 Hz, -CH-5′, -CH-5″), 1.18 (6H, *d*, J = 6.0Hz, -CH_3_-6′ and–CH_3_-6″), 1.01 (6H, *d*, J = 6.0Hz, -CH_3_-7′ and–CH_3_-7″). ^13^C NMR (DMSO-d6, 75 MHz): δ 127.2 (C-1), 101.1 (C-2), 157.3 (C-3), 93.2 (C-4), 97.5 (C-5), 164.1 (C-6), 102.1 (C-7), 131.2 (C-8), 179.9 (C-9, C = O), 162.6 (C-10), 115.1 (C-11), 156.1 (C-12), 113.2 (C-13), 71.3 (C-1′, C-1″), 66.0 (C-2′, C-2″), 49.4 (C-3′, C-3″), 46.7 (C-5′, C-5″), 24.66 (C-6′, C-6″), 23.8 (C-7′, C-7″). TOF MS ES+ (70 eV): *m/z* 458 (M+, C_25_H_34_N_2_O_6_) (40), 414 (species-a, base peak) (100), 343 (species-b) (8) or 343 (species-c) (8), 243 (species-d) (43), 185 (species-e) (63).

**3, 6-Di–[3-(iso-propyl-amino)-2-hydroxy-propoxy] -1-hydroxy xanthone (2):** Obtained the product (2b) as reddish brown semisolid, soluble in water. m.p. 98–99°C, Rf: 0.31 [Benzene: Methanol (8:2)], yield: 72.4%. UV (λ max CH_3_OH): 307, 260, 239, 226, 212 nm. IR: n max (KBr): 3400 (NH, OH), 2950 (CH_3_), 2850 (CH_2_), 1654 (C = O), 1545 (C = C and N-H), 1446, 1380, 1287, 1179, 1106 (N-H), 1016 (C-O, alcoholic), 827 cm^-1^. ^1^H NMR (DMSO-d6, 400 MHz): δ 8.01 (1H, *d*, J = 2.0 Hz, H-2), 8.15 (1H, *d*, J = 2.0 Hz, H-4), 8.45 (^1^H, *d*, J = 2.0 Hz, H-5), 8.16 (^1^H, *d*, J = 8.5 Hz, H-8), 8.15 (1H, *dd*, J = 8.5, 2.0 Hz, H-7), 3.88 (4H, *m*, -OCH_2_-1′ and -OCH_2_-1″), 3.24 (2H, 2 pentets, W1/2 = 17.5 Hz, -CH-2′ and CH-2″), 2.80 (4H, *m*, -CH_2_-3′ and -CH_2_-3″), 2.50 (2H, *s* N-H- 4′, and N-H-4″), 1.84 (2H, septet W1/2 = 17.5 Hz, -CH-5′, -CH-5″), 1.18 (6H, *d*, J = 6.0Hz, -CH_3_-6′ and–CH_3_-6″), 1.01 (6H, *d*, J = 6.0Hz, -CH_3_-7′ and–CH_3_-7″). ^13^C NMR (DMSO-d6, 75 MHz): δ 165.0 (C-1), 101.1 (C-2), 157.3 (C-3), 93.2 (C-4), 97.5 (C-5), 164.1 (C-6), 102.1 (C-7), 131.2 (C-8), 179.9 (C-9), 162.6 (C-10), 115.1 (C-11), 156.1 (C-12), 113.2 (C-13), 71.3, 70.6 (C-1′, C-1″), 67.8, 66.0 (C-2′, C-2″), 49.9, 49.4 (C-3′, C-3″), 47.2, 46.7 (C-5′, C-5″), 24.66 (C-6′, C-6″), 23.8 (C-7′, C-7″). TOF MS ES+ (70 ev): *m/z* 474 (M+, C_25_H_34_N_2_O_7_) (18), 434 (species-a) (12), 415 (species-b) (17), 414 (species-c, base peak) (100), 361 (species -d) (50), 300 (species-e) (15), 243 (species-f) (17), 185 (species-g) (8).

### Antihypertensive screening

#### Experimental animals

Albino Wistar rats of either sex, weighing 180-210g, were obtained from animal house, Jamia Hamdard, Hamdard Nagar, New Delhi 110062 and kept in separated cages under standard environmental conditions of temperature 20 to 30ºC and humidity and were provided with standard rat chow and water *ad libitum*. This study was carried out in strict accordance with the recommendations in the Guide for the Care and Use of Laboratory Animals of the institutional animal ethical committee regulations of Jamia Hamdard (IAEC-Jamia Hamdard). The protocol was approved by the Committee on the Ethics of Animal Experiments of Jamia Hamdard University (Reg. No.: 173/GO/Re/S/2000 /CPCSEA). All experiment was performed under diethyl ether anesthesia, and all efforts were made to minimize suffering. Cervical dislocation was used as method of sacrifice.

#### Drugs

The standard drugs atenelol and propranolol and the methyl prednisolone acetate were procured from sigma chemicals.

#### Instruments

The change in systolic, diastolic and the mean blood pressure before and after the treatment of the drugs was measured on a Digital LE-S100 Blood Pressure Meter in Tail-cuff method non-invasively at full cautious state of the animals and compared with the standard drugs.

#### Method

The standard drug methyl prednisolone acetate was administered by intraperitonial route at a single dose of 1 mg/ kg (body weight) to a group of rats. The control group was given only saline (NaCl, 0.9%) at the rate of 5 ml/ rat. Another group of rat was administered standard drug methyl prednisolone acetate intraperitoinally at a single dose of 1 mg/ kg followed by the administration of the test compounds intraperitonially and orally at a single dose of 10 mg/ kg each and reference drugs propranolol and atenolol 35 mg/ kg respectively. The rise in mean arterial BP and heart rate was observed at once appeared on digital pressure meter at the interval of each 15 minutes and the average change in mean blood pressure and heart rate was also calculated and recorded [12].

### Toxicity study

The novel compounds were also evaluated for their toxicity. Liver function test [13]—Determination of Serum Glutamic Oxaloacetic Transaminase (SGOT); Serum Glutamic Pyruvate transaminase (SGPT); Alkaline Phosphatase ALP; was carried out after intraperitonial administration of single dose i.e. 10mg/ kg of all the tested compounds. Other toxicity studies such as kidney function test [14,15]—determination of level of urea and creatinine, cardiac function test [16, 17]—determination of lactate dehydrogenase (LDH), lipid function test [18–21]—determination of triglyceride (TG),Acetylcholine esterase assay—determination of level of acetylcholine (ACH), Glutathione (GSH) assay, catalase assay, Superoxide dismutase (SOD) estimation [22] and Thiobarbituric acid reactive substances (TBARS) assay [23] were carried out after oral administration to rats at doses of 25, 50, and 100mg/kg at Deshpande Laboratories Pvt. Ltd. An ISO 9001:2008 Certified Drug Testing Laboratory CPCSEA Approved: 1410/c/11/CPCSEA, Bhopal, M.P. India.

### Molecular docking study

The docking simulations of all the compounds were performed using Maestro, version 9.4 implemented from Schrodinger software suite. The ligands were sketched in 3D format using build panel and were prepared for docking using ligprep application. The protein for docking study was taken from protein data bank (PDB ID: 4BVN) and prepared by removing solvent, adding hydrogen and further minimization in the presence of bound ligand using protein preparation wizard. Grids for molecular docking were generated with bound co-crystallized ligand, for the validation of docking parameters the standard ligand (propranolol) was re-docked at the catalytic site of protein and the RMSD between co-crystal and re-docked pose was found to be 0.255 A. All the three synthesized compounds were docked using Glide extra-precision (XP) mode, with up to three poses saved per molecule.

## Results and discussion

### Antihypertensive activity study

**Fig 2:**
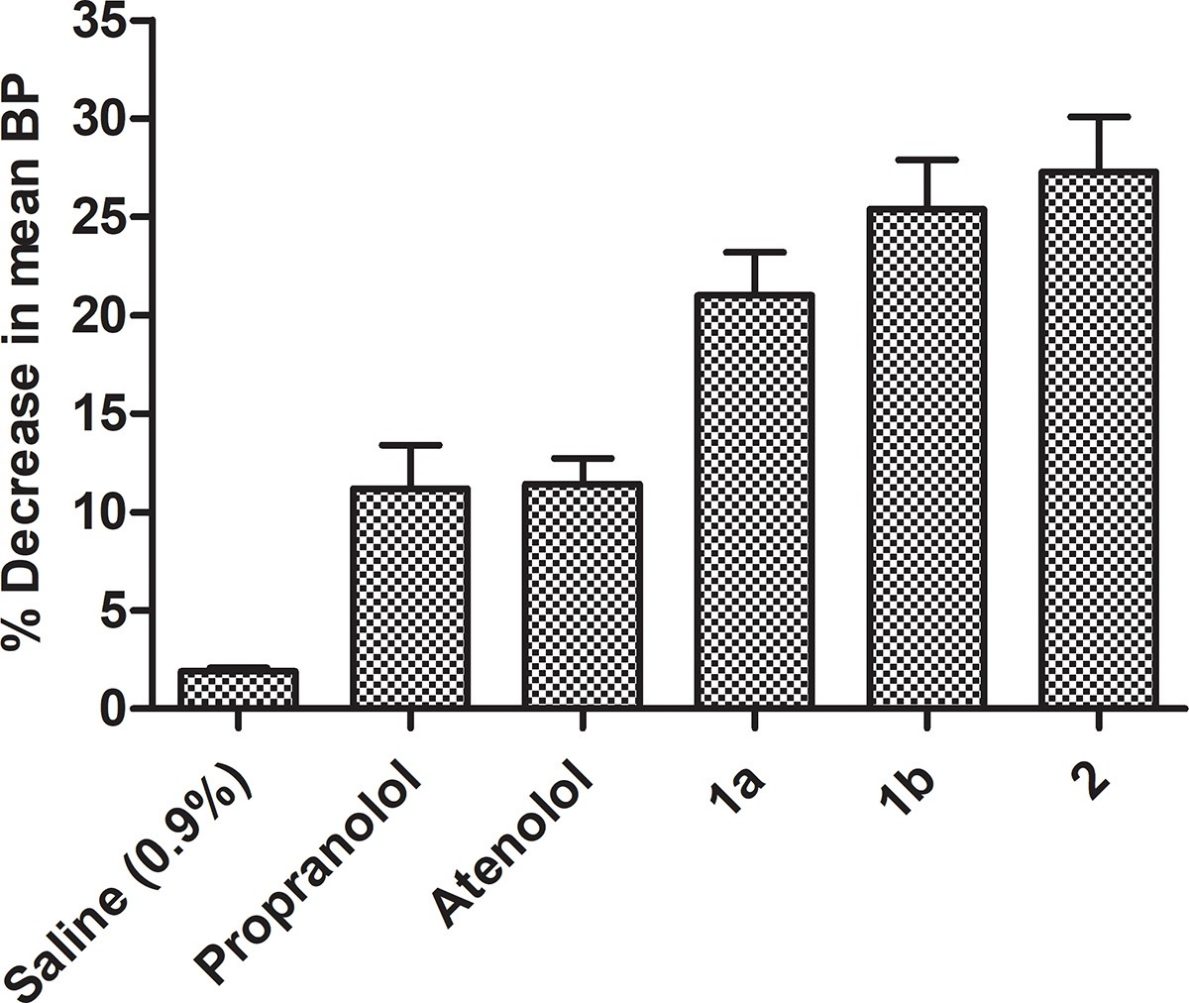
Activity profile curve (i.p. route).

**Fig 3:**
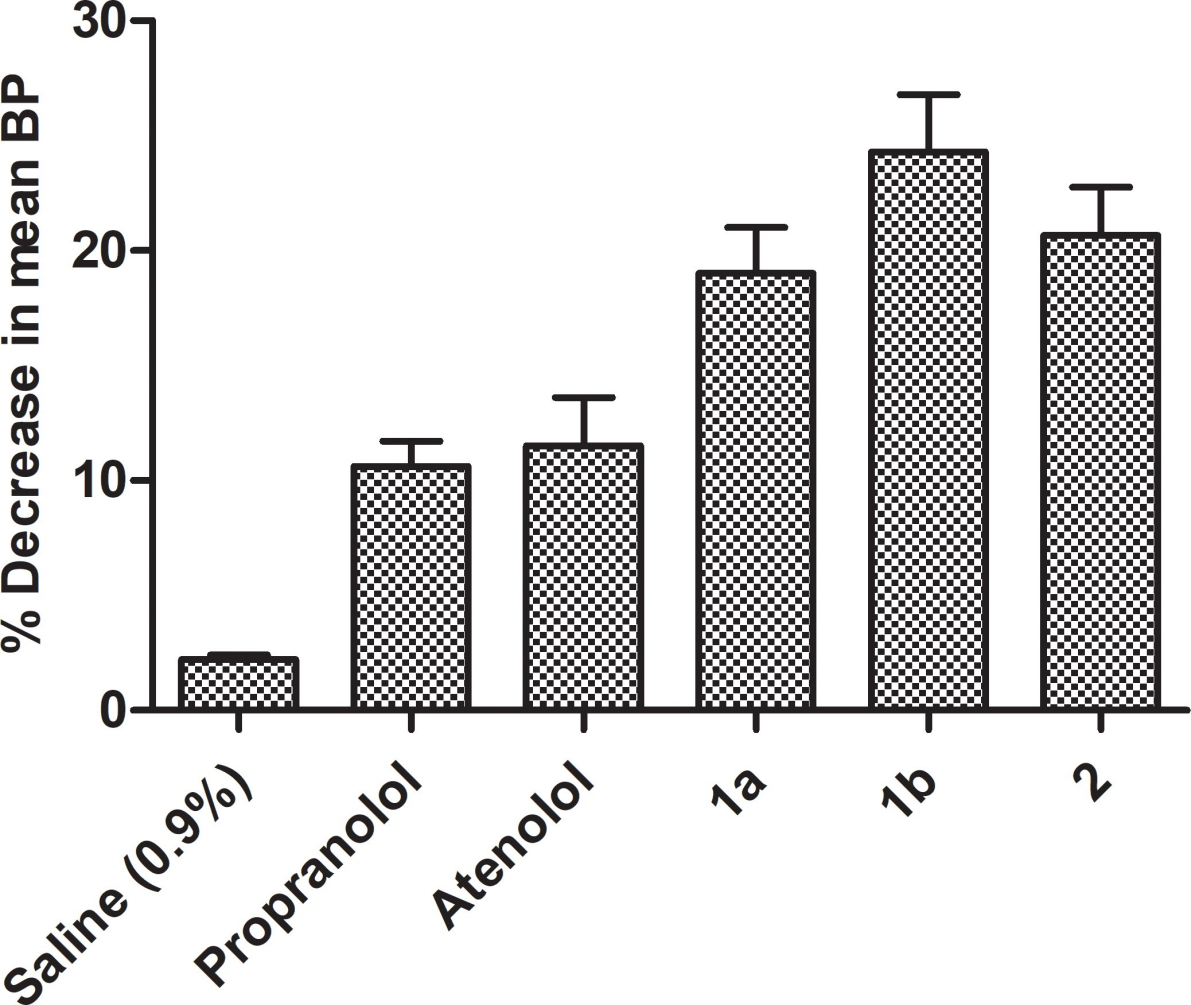
Activity profile curve (oral route).

Xanthone derivatives reduce the blood pressure by blocking calcium channel and *β*receptors [24]. Synthesis of xanthonoxypropanolamines were carried out by first reaction of 2, 4-dihydroxy benzoic acid with appropriate phenol (resorcinol, phloroglucinol) in presence of freshly fused zinc chloride and POCl_3_ which gave di and tri-hydroxy xanthones. The solution of hydroxyl xanthones in an aqueous ethanol containing sodium hydroxide and epichlorhydrin was refluxed to give epoxy compounds (3-aryloxy-2, 3-epoxy propane) in good yield (78–81%), and then the ethanolic solution of the epoxy derivatives were refluxed in large excess of different type of alkyl/ aryl amines which yielded the xanthonoxypropanolamines (Fig 1). The xanthonoxypropanolomines were purified by recrystallization with appropriate solvent. IR, ^1^H-NMR, ^13^C NMR and MASS spectral data of all the synthesized compounds were consistent with the assigned structures. All the synthesized compounds were screened for the antihypertensive activity and toxicity of novel compounds. The result (Tables 1 and 2 and Figs 2 and 3) showed that the compound **1a, 1b** and **2** have greater activity as these compound showed **11.30%, 18.8%** and **12.6%** reduction in systolic blood pressure and **21.00%, 25.4%** and **27.30%** in mean blood pressure respectively at the doses of **10 mg/kg** while the standard drug Propranolol and Atenolol showed only **11.70%** and **11.76%** reduction in systolic blood pressure and **11.20%** and **11.42%** in mean blood pressure respectively at the doses of **35 mg/kg** on i.p administration of drugs. After oral administration of drug, the percentages of reduction in systolic blood pressure were **12.00%, 19.20%** and **11.34%** and **19.00%, 20.48%** and **20.66%** in mean blood pressure respectively by **1a, 1b** and **2** while percentages of reduction in systolic blood pressure were **11.48%** and **10.71%** and in mean blood pressure **10.60%** and **11.52%** by standard drug Propranolol and Atenolol respectively.

**Table 1:** Average effect of standard drugs and test compounds (i. p. route).

Compounds	Dose	Pretreated BP Induced (mmHg)		After1 hr of Treatment (mmHg)		Change in BP (mmHg)		% Decrease in BP (mmHg)	
		ASBP	AMBP	ASBP	AMBP	ASBP	AMBP	Systolic	Mean
Saline (0.9%)	5 ml	115 ± 7.8	104 ± 1.1	115 ± 6.8	102 ±9.1	00 ± 2.0	02.0 ± 2.0	00 ± 2.0	1.9 ± 0.2
Propranolol	10 mg	111.5 ±8.5	96 ± 10.6	98.0 ± 7.0	085 ± 7.2	13.2 ± 2.2	11.0 ±3.0	11.70 ± 2.9	11.20± 1.2
Atenolol	10 mg	119.0 ±7.8	105.0 ± 9.0	105 ± 6.0	093 ± 7.5	14.5 ± 2.0	12.0 ±1.7	11.76± 1.6	11.42 ± 1.3
(1a)	3 mg	115.5 ± 1.3	100.5 ± 1.7	103.1 ± 1.0	080 ± 1.2	13.5 ± 1.4	21.0 ± 2.7	11.30 ± 1.7	21.00 ± 2.2
(1b)	2.8mg	117.0 ± 4.7	104.6 ± 4.5	095 ± 2.6	078 ± 4.0	22.0 ± 2.5	26.5 ±2.1	18.80 ± 2.5	25.40 ± 2.5
(2)	3 mg	111.0 ±2.2	96.4 ±2.8	097 ± 1.4	070 ± 2.5	14.5 ± 3.2	26.0 ±2.0	12.60 ± 2.0	27.30± 2.8

BP = Blood pressure, ASBP = Arterial Systolic Blood Pressure, AMBP = Arterial Mean Blood

Pressure

**Table 2:** Average effect of standard drugs and test compounds (oral route).

Compounds	Dose	Pretreated BP- Induced (mmHg)		After1 hr of Treatment (mmHg)		Change in BP(mmHg)		% Decrease in BP(mmHg)	
		ASBP	AMBP	ASBP	AMBP	ASBP		AMBP	
Saline (0.9%)	5 ml	113.0 ± 2.4	103.0 ± 1.8	109.0 ± 1.7	100.5 ± 1.8	04.0 ± 0.7	02.5 ± 1.5	03.00 ± 1.6	02.20 ± 0.2
Propranolol	16 mg	112.0 ± 3.9	097.0 ± 2.1	096.5 ± 2.6	081.5 ± 1.7	15.5 ± 1.3	12.0 ± 0.4	11.48 ± 0.5	10.60 ± 1.1
Atenolol	15 mg	117.5 ± 1.9	105.0 ± 3.6	102.5 ± 1.2	091.0 ± 1.6	15.0 ± 0.7	14.0 ± 2.0	10.71± 1.8	11.52± 1.2
(1a)	3.5 mg	116.5 ± 3.2	101.0 ± 1.4	099.5 ± 1.9	078.0 ± 1.1	17.0 ± 1.3	23.0 ± 0.3	12.00 ± 0.1	19.00 ± 2.0 1 2.0
(1b)	3.5 mg	117.0 ± 4.5	102.5 ± 3.3	091.0 ± 3.7	073.5 ± 2.3	26.0 ± 0.8	29.0 ± 1.0	19.20 ± 1.0	24.48 ± 2.5
(2)	4 mg	111.5 ± 2.8	097.5 ± 2.2	096.5 ± 1.6	072.5 ± 1.2	15.0 ± 1.2	25.0 ± 1.0	11.34 ± 1.3	20.66 ± 2.1

BP = Blood pressure, ASBP = Arterial Systolic Blood Pressure, AMBP = Arterial Mean Blood Pressure

### Toxicity study

**Fig 4:**
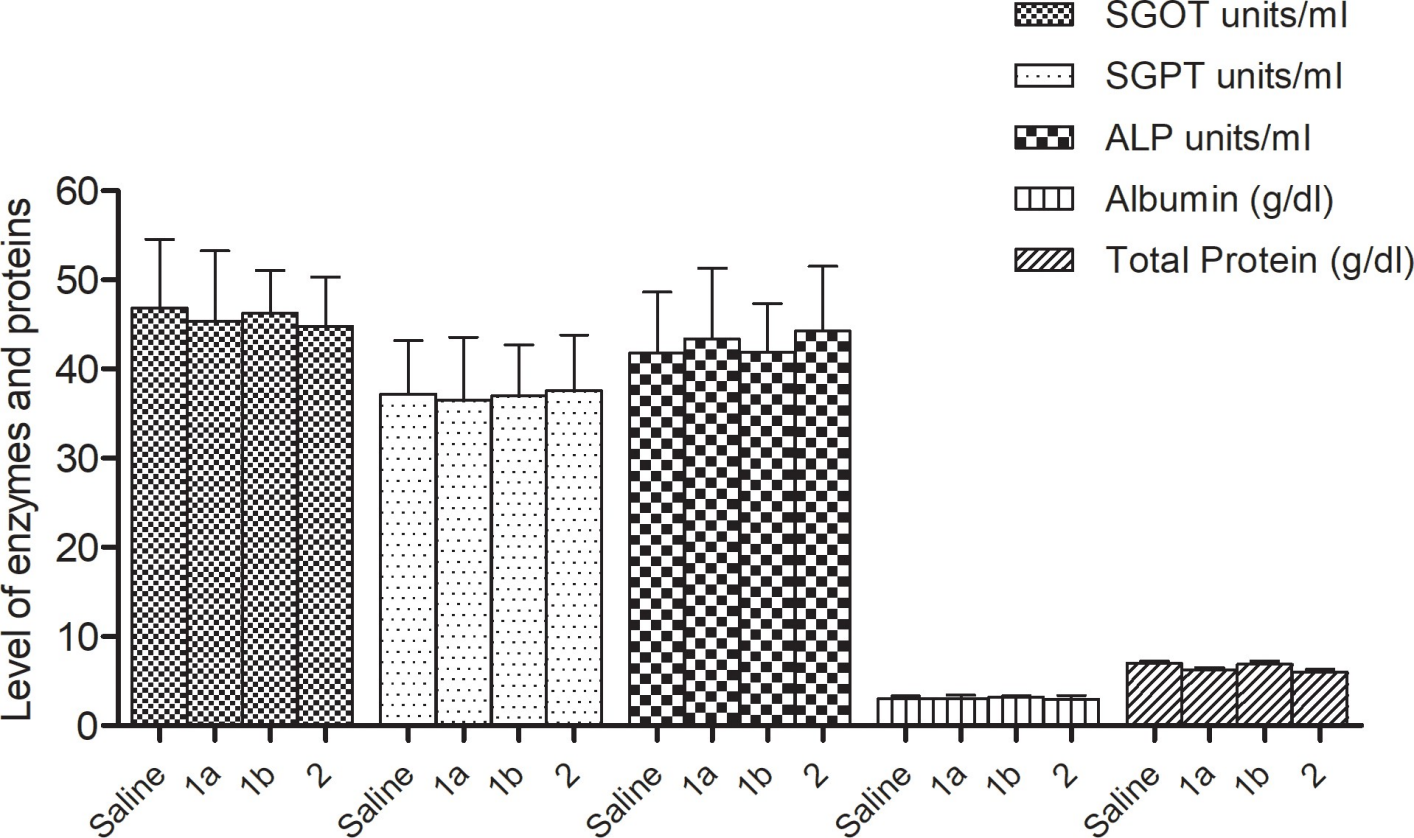
Graphical representation of SGOT, SGPT, ALP, Albumin, total protein (Liver function test—i.p. route).

Also these compounds **1a, 1b** and **2** had almost equal or less toxicity profile as the data shown in Tables **3** and **4** and Fig 4 clearly indicates the level of different biochemical parameters in comparison to standard drug Propranolol and Atenolol. The compounds did not exhibit any toxicity during testing of antihypertensive activity, as no death of any rat occurred during antihypertensive screening study and the level of different biochemical parameters (urea, creatinine, triglycerides, LDH, GSH, SOD, catalase, TBARS and liver enzymes) were almost equal or less in comparison with standard drugs atenolol and propranolol.

**Table 3:** Comparison of liver function: Enzyme activity, Alkaline Phosphatase and proteins level.

Groups (n = 5)	Treatment	i. p. Single Dose	SGOT units/ml	SGPT units/ml	ALP units/ml	Albumin (g/dl)	Total Protein (g/dl)
1	Normal	—	46.83 ±7.70	37.18 ±5.98	41.79 ± 6.85	3.05 ± 0.26	6.97 ± 0.27
2	(1a)	3.5 mg	45.37 ±7.91	36.49 ±7.1	43.39 ± 7.94	3.02 ± 0.39	6.28 ± 0.25
3	(1b)	3.5 mg	46.27 ±4.80	36.95 ±5.73	41.9 ± 5.44	3.19 ± 0.15	6.86 ± 0.37
4	(2)	4 mg	44.85 ±5.49	37.57 ±6.27	44.28 ± 7.22	2.96 ± 0.43	5.98 ± 0.33

SGOT = Serum Glutamic Oxaloacetic Transaminase; SGPT = Serum Glutamic Pyruvate transaminase; ALP = Alkaline Phosphatase; i. p. = Intraperitonially; P > 0.05 non-significant. Values are mean ± S.D. (n = 5). ANOVA followed by Dennett's test

**Table 4:** Level of different parameters in serum found at Tmax after oral administration of test compounds and standard drugs atenolol [25–29] and propranolol [30–33].

S. No.	Test	Control group	(1b)			(2)			(1a)			Atenolol		Propranolol	
			mg/kg			mg/kg			mg/kg			mg/kg		mg/kg	
			25	50	100	25	50	100	25	50	100	W/O	With	W/O	With
1	Urea (mg/dl)	17.6	19.90	19.43	29.00	18.80	26.40	23.53	25.00	23.50	23.53	180±98*	229±111*	16.5±1.1#	16.0 ± 0.4#
2	Creatinine (mg/dl)	0.56	0.56	0.56	0.56	0.4	0.4	0.56	0.73	0.73	0.73	1.6±0.1**	1.6±0.18**	0.45±0.01$	0.42± 0.01$
3	TG (mg/dl)	98.76	101.00	76.53	104.30	97.66	103.23	107.66	101.00	101.00	118.80	129.83±6.04***	160.83±6.97***	114±7.0^d^	64.1± 4.2^d^
4	ACH (mU/ml)	10	10	10	10	10	10	10	10	10	10	NR		NR	
5	LDH (μU)	123.00	128.53	61.90	184.10	195.20	117.43	89.66	123.00	173.00	89.66	a737.22 + 29.61	a759.0 3 + 10.30	112±8^	131±12^
6	GSH (μM)	4.72	6.05	5.03	7.14	5.63	7.01	5.39	7.44	7.44	5.33	bNo changes up to10 μM		bglutathione losses were attenuated 15 to 80%, with EC(50) = 3.1 μM	
7	SOD(U/ml)	2.51	3.17	2.64	2.84	2.84	2.84	2.78	2.52	2.51	2.84	12.27+2.65^a^	12.79+0.86^a^	2.51±0.08^c^	4.93±0.04^c^
8	Catalase (μM)	118.94	96.27	99.11	121.77	113.27	121.77	107.61	113.27	101.94	101.94	16.96+0.86^a^	36.92+0.81^a^	3.26±0.05^c^	4.96±0.08^c^
9	TBARS (μM)	24.01	14.62	15.68	16.13	13.40	15.98	14.62	15.53	14.62	16.59	20.33+1.45^a^	17.33+0.88^a^	@No changes at 10 mg/kg	

W/O = without treated, W/T = treated, NR = not reported

* = Level of blood urea nitrogen (BUN) at 80mg/kg per day in renal mass reduced rats

** 10 mg/kg daily up to 6 weeks

*** = in streptozotocin induced diabetic rats at dose of 10mg/kg up to 8 weeks

# (level of urea nitrogen)

$, ^ (U/L) = in plasma with propranolol treatment at 10mg/kg

@ = in cyclosporine A—induced oxidative stress and renal dysfunction

a = in heart tissue homogenate (LDH in serum- U/L) in isoproteronol induced myocardial damage with atenolol treatment at 6mg/kg (U/mg protein)

b = in iron overloaded and oxidative stressed endothelial cells (ECs)

c = in heart tissue homogenate at 10 mg/kg propranolol treatment for 7 days U/mg protein)

d = in plasma at 25 mg/kg.

### Molecular docking study

**Fig 5:**
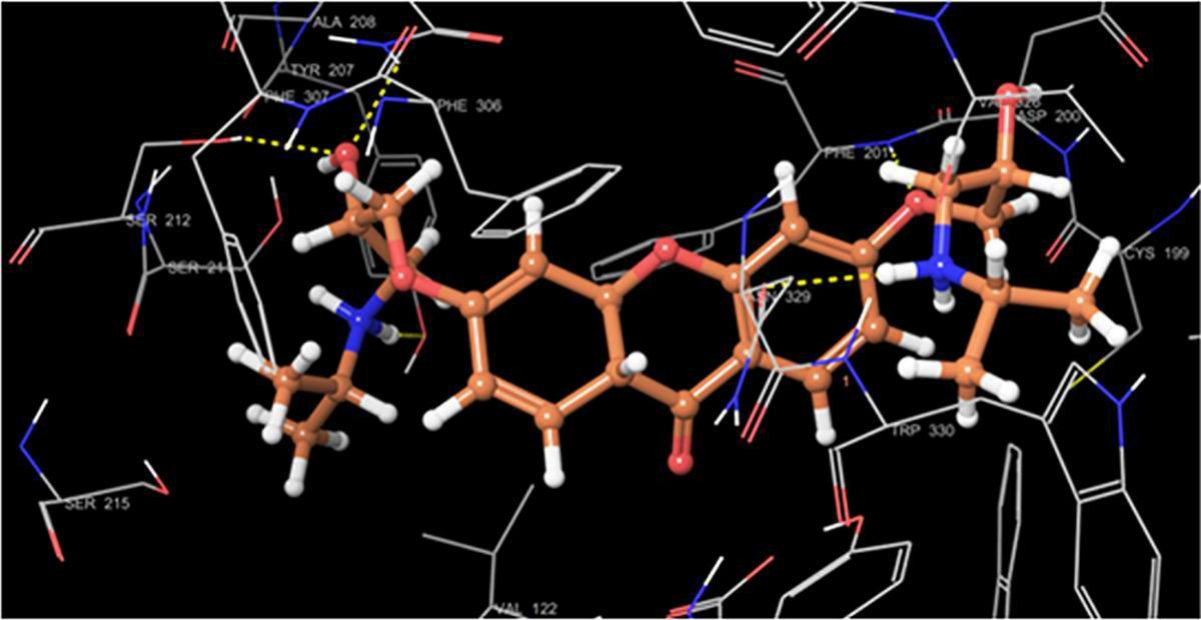
The binding mode of 1b (grey) in the *β*1 adrenoreceptor active site.

**Fig 6:**
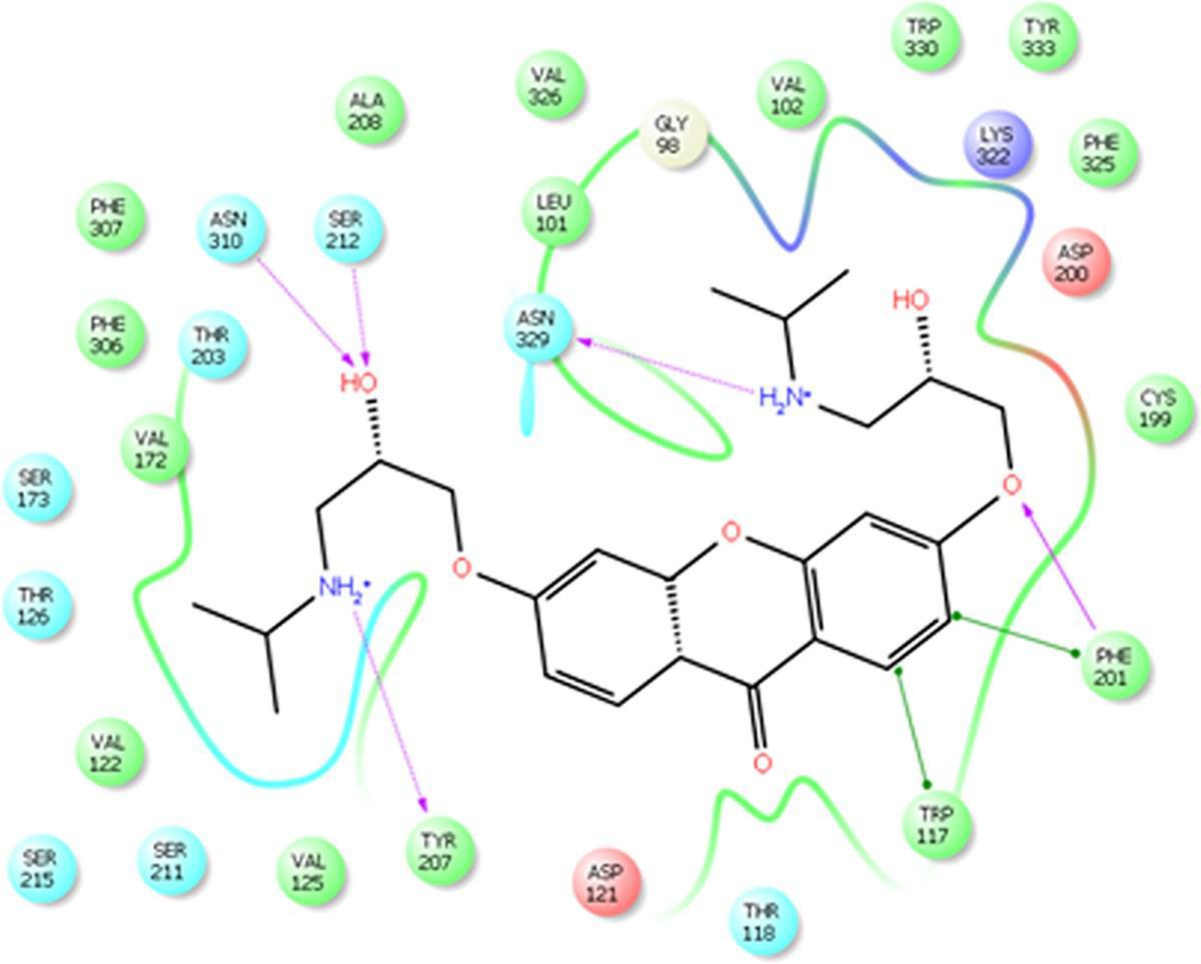
Ligplot of 1b with *β*1 receptor active site.

**Fig 7:**
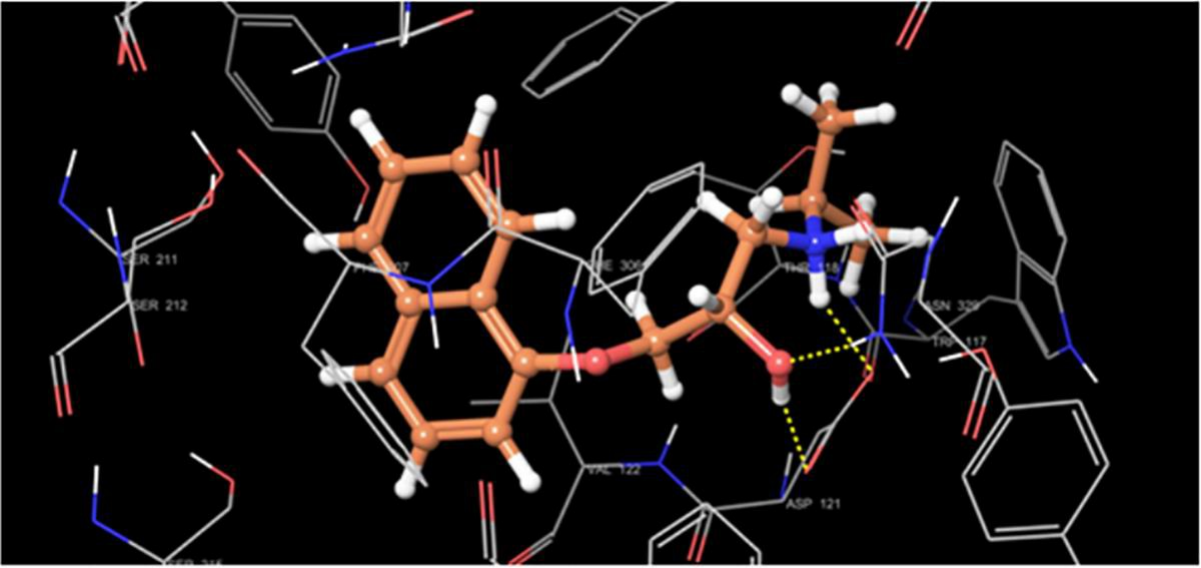
Binding pattern of propranolol with *β*1.

**Fig 8:**
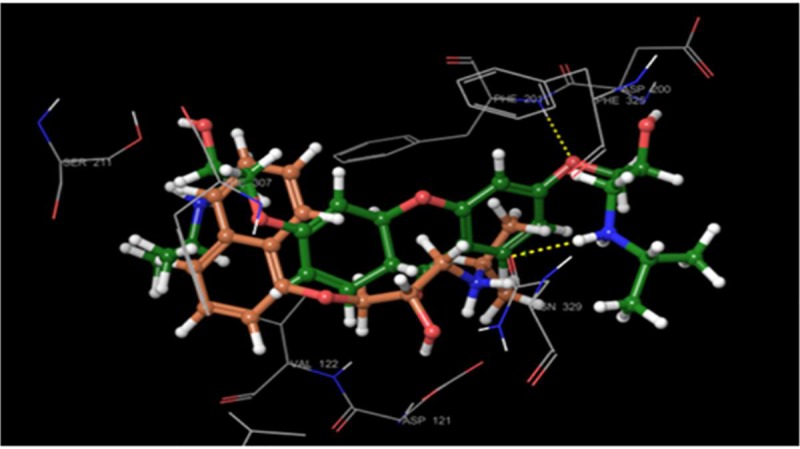
Comparison of 1b (green) and propranolol ligand (grey) binding mode in the *β*1 active site.

Molecular docking on all the three synthesized compounds was performed against *β*1 adrenoreceptor and the docking studies revealed a common binding orientation of all the synthesized compounds in the catalytic binding pocket of *β*1 adrenoreceptor. The amino alkyl moiety plays an important role in the binding, as the carbonyl oxygen, nitrogen atom are involved in hydrogen bonding interactions with amino acid residue Tyr207, Ser212, Asn310, Phe201, Asn329 at the catalytic site as shown in Fig 5 and ligplot (Fig 6). A pi-pi stacking interaction was also observed between phenyl ring of the xanthone moiety and the phenyl ring of the amino acid residue Phe201 and Tyr207 in all the compounds. In addition, the phenyl group moiety showed hydrophobic interaction with β1 adrenoreceptor protein Tyr333, Phe201, Ser173 and Phe307 amino acid residues. The comparison of binding pose of compound 1b and propranolol with *β*1 adrenoreceptor protein was also reported (Figs 7 and 8).

For better understanding of binding mode of amino alkyl substituted xanthone derivatives at the molecular level, we carried out molecular docking simulations of all the three synthesized molecules at the β1 adrenoreceptor catalytic ligand binding site. The docking score for the compound **1b** was found **-9.1** while for compound **1a** and **2** were found **-8.7** and **-8.6** respectively.

## Conclusions

It was observed during the studies that these novel compounds i.e. 1a, 1b, and 2 have greater antihypertensive activity in comparison to standard drugs Propranolol and Atenolol, as these compounds showed greater percentage of reduction in both systolic and mean blood pressure. The compounds did not exhibit any toxicity during study, as no death of any rat occurred during antihypertensive screening study and the level of different biochemical parameters (urea, creatinine, etc.) were almost equal or less in comparison with standard drugs atenolol and propranolol. The binding of these synthesized compounds with beta adrenoceptors were better than Propranolol, as the docking score for these compounds were found to be **-9.1** for 1b while for compound **1a** and **2** were found to be **-8.7** and **-8.6** respectively. Thus, it concludes that these compounds can be used as lead molecules for future investigations.
